# A Prospective Study among Patients Presenting at the General Practitioner with a Tick Bite or Erythema Migrans in the Netherlands

**DOI:** 10.1371/journal.pone.0064361

**Published:** 2013-05-16

**Authors:** Agnetha Hofhuis, Tineke Herremans, Daan W. Notermans, Hein Sprong, Manoj Fonville, Joke W. B. van der Giessen, Wilfrid van Pelt

**Affiliations:** 1 Epidemiology and surveillance unit, Centre for Infectious Disease Control Netherlands, National Institute for Public Health and the Environment, Bilthoven, The Netherlands; 2 Laboratory for infectious diseases and perinatal screening, Centre for Infectious Disease Control Netherlands, National Institute for Public Health and the Environment, Bilthoven, The Netherlands; 3 Laboratory for zoonoses and environmental microbiology, Centre for Infectious Disease Control Netherlands, National Institute for Public Health and the Environment, Bilthoven, The Netherlands; University of Minnesota, United States of America

## Abstract

**Background:**

We performed a nationwide prospective study on the transmission risk for *Borrelia* to humans, investigating symptoms and serology at enrolment and three months after tick bites, and after standard treatment for erythema migrans (EM). Aiming to quantify the infection risk at point of care by physicians, we explored risk factors such as tick testing for *Borrelia* and assessment of the duration of the tick's blood meal.

**Methods and Findings:**

Questionnaires, blood samples and ticks from patients who consulted one of 307 general practitioners for tick bites (n = 327) or EM (n = 283) in 2007 and 2008, were collected at enrolment and three months later at follow-up. *Borrelia burgdorferi* sensu lato DNA was detected in 29.3% of 314 ticks, using PCR/reverse line blot and real-time PCR on the OspA gene. Seroconversion in C6 ELISA, IgM or IgG immunoblots for *Borrelia*-specific antibodies was observed in 3.2% of tick bite cases. Fourteen tick bite cases had evidence of early *Borrelia* infection, of which EM developed among seven cases. The risk of developing EM after tick bites was 2.6% (95%CI: 1.1%–5.0%), and the risk of either EM or seroconversion was 5.1% (95%CI: 2.9%–8.2%). Participants with *Borrelia*-positive ticks had a significantly higher risk of either EM or seroconversion (odds ratio 4.8, 95%CI: 1.1–20.4), and of seroconversion alone (odds ratio 11.1, 95%CI: 1.1–108.9). A third (34%) of the cases enrolled with EM did not recall preceding tick bites. Three EM cases (1%) reported persisting symptoms, three months after standard antibiotic treatment for EM.

**Conclusions:**

One out of forty participants developed EM within three months after tick bites. The infection risk can be assessed by tick testing for *Borrelia* at point of care by physicians. However, further refining is needed considering sensitivity and specificity of tick tests, accuracy of tick attachment time and engorgement.

## Introduction

Lyme borreliosis is caused by different *Borrelia* species from the *Borrelia burgdorferi* sensu lato group (hereafter referred to as *Borrelia*), which in Europe is transmitted by the tick *Ixodes ricinus*. The most common clinical manifestation of Lyme borreliosis is erythema migrans (EM), a characteristic rash expanding from the site of the tick bite, which may appear some days to weeks following infection, and is sometimes accompanied by systemic flu-like symptoms. Late and more serious Lyme borreliosis can present as a multi-systemic disease with skin, neurological, cardiac and musculoskeletal manifestations. [1] In the Netherlands, a repeated retrospective study among general practitioners has shown a continuing and strong increase in consultations for tick bites and for EM between 1994 and 2009 [2]–[4]. The increasing number of tick bites, adding up to 1.5 million people with a tick bite in 2009 [4], poses a progressive threat to public health.

To aid the development of prevention strategies against Lyme borreliosis, knowledge of the epidemiology and risk factors are essential. Specifically, the understanding and quantification of an individual's risk for *Borrelia* infection and developing symptomatic Lyme borreliosis after a tick bite would be of great value to establish the usefulness of antibiotic prophylaxis after a tick bite. The individual risk for *Borrelia* infection depends on several factors, one of these being the tick infection rate with *Borrelia*, which tends to be heterogeneous over space and time [5], [6]. Another is the transmission rate of *Borrelia* from ticks to humans, which is affected by the tick attachment time. According to experimental data, *Borrelia* transmission does not occur at the beginning of the blood uptake. The transmission efficiency increases with the duration of the blood meal, as described for the North American vector *Ixodes scapularis* infected with *Borrelia burgdorferi* sensu stricto. Nymphal *Ixodes scapularis* ticks require attachment to the host for at least 24 hours before transmission of *Borrelia* starts, and a high level of transmission is reached after 48 hours of attachment [7], [8]. In Europe however, transmission of *Borrelia* during the first 24 hours of *Ixodes ricinus* attachment has also been reported [9], [10]. According to North American studies, prophylactic antibiotic treatment after a tick bite can prevent Lyme borreliosis [11], provided that the tick bite is not overlooked, which is the case for one third, up to two thirds of tick bites [12], [13]. A major disadvantage of treating all detected tick bites prophylactically, would be the high number needed to treat (NNT) to prevent one new case of Lyme borreliosis. Therefore we aim to explore to what extent the NNT can be reduced, using tick-screening instruments for general practitioners to predict an individual's risk of *Borrelia* infection after each tick bite. Such tick-screening instruments include tick testing for infection with *Borrelia*, and assessment of the duration of the tick's blood meal, measured as self-estimated hours of tick attachment time or measured as degree of engorgement of the tick.

The issue of transmission rate of the pathogen from ticks to humans in Europe has been addressed by studies in Switzerland [14], [15], and Sweden [16]. In the Netherlands, a study was performed in 2006 on Ameland, one of the Northern Wadden islands [17]. However, these results were not considered representative of the whole of the Netherlands, due to the small sample size (n = 146) of this study, and as tick infection rates with *Borrelia* tend to be spatially and temporally heterogeneous. Here, we report the results of a nationwide prospective study among patients who consulted a general practitioner for a tick bite or EM. Tick bite patients were followed-up after three months to investigate the transmission risk for *Borrelia* to humans in the Netherlands, to gain insight in risk factors for tick bites and for *Borrelia* infection, exploring tick-screening instruments to predict an individual's risk of *Borrelia* infection after each tick bite, and to explore associations with symptoms and serology. EM patients were followed-up after three months to investigate symptoms and serology after standard antibiotic treatment, and to gain insight in risk factors for *Borrelia* infection.

## Materials and Methods

### Study design

Out of two thousand invited general practitioners in areas with a high incidence of tick bites, as identified in an earlier study [3], 307 general practitioners from all twelve provinces of the country agreed to enroll patients into our prospective tick bite study. Between May 2007 and December 2008, patients who consulted a cooperating general practitioner for a recent EM or tick bite, preferably still having the tick, were invited to participate. Patients were not eligible for participation if they were younger than six years of age, and if the tick bite had occurred outside the Netherlands. At enrolment at the general practitioner, participants received the first set of study materials, containing a brochure about the study, an informed consent form, a baseline questionnaire, and materials for collection and mailing of baseline blood samples and removed ticks. Ticks removed from the skin were sent to our study laboratory at the RIVM by regular mail, using a small tube with 70% ethanol. Two tubes of blood, 7 ml in a serum tube and 5 ml in an EDTA tube, were collected at regular medical posts for blood withdrawal and sent to our study laboratory at the RIVM. The first questionnaire inquired about baseline data such as the location of tick bites and EM on the body, probable duration of tick attachment, in which area and during which activity the tick bite was possibly contracted, techniques for tick removal, use of antibiotics, symptoms, and history of tick bites and Lyme borreliosis. The questionnaire also inquired about risk behaviour, knowledge of tick bites and Lyme borreliosis, and attitudes towards preventive measures. Three months after enrolment, the participant received the second set of study materials, containing a follow-up questionnaire and materials for collection and mailing of a second blood sample. The follow-up questionnaire inquired about new tick bites, development of Lyme borreliosis, symptoms and the use of antibiotics during the period between the baseline and follow-up questionnaires. For epidemiological analysis, participants were divided into a group enrolled with an EM and a group enrolled with a tick bite. To support correct classification of EM cases, the general practitioners received an additional confirmation questionnaire for each case who reported an EM at baseline or in the follow-up questionnaire. Development of clinical Lyme borreliosis, as reported in the participant's questionnaire, was only taken into account as an outcome measure in this study, if confirmed by the general practitioner through this additional questionnaire. After having enrolled a case, the general practitioner invited a control person to fill out a questionnaire similar to the baseline case questionnaire, to enable comparison of cases and control persons with regard to risk behaviour, knowledge of tick bites and Lyme borreliosis, and attitudes towards preventive measures (not reported in this article). Eligible controls were patients who visited the same general practitioner for reasons other than a tick bite or Lyme borreliosis, preferably of the same gender and age as the corresponding case. General practitioners were additionally requested to register the number of consults for tick bites and EM on a scoring card on a weekly basis for the years 2007 and 2008. To keep the general practitioners informed and alert on the study, we sent three-monthly newsletters. The study protocol (number 07-032/K) was approved by the medical ethics committee of the University Medical Centre in Utrecht, the Netherlands. All participants gave written informed consent.

### Tick analyses

After arrival at the laboratory, ticks were stored at −20°C in ethanol until microscopic examination was performed to determine tick species, stage and gender, using standard keys [18]. The degree of engorgement of the tick was categorized as unengorged, partially engorged, or fully engorged. Total DNA extraction from ticks, amplification by PCR, reverse line blotting (RLB) for *Borrelia* species identification were performed as described.[19], [20] In addition, the presence of *Borrelia* in ticks was also determined using a real-time PCR amplification on the OspA gene.[21] Individual test results of the tick analyses were not reported to the participants or their physicians.

### Serological analyses

Paired serum samples (the baseline and consecutive serum sample) from the same case were tested simultaneously for *Borrelia*-specific antibodies using a commercially available C6 peptide ELISA and in house IgM and IgG immunoblots. The C6 ELISA was performed according to the manufacturer's instruction [Immunetics, Inc. Cambridge, Mass. USA]. Results were scored as negative (ELISA index score, <0.90), borderline (0.90 to 1.09), or positive (≥1.10). The C6 ELISA has a reported sensitivity of 23 to 90% in EM patients [22]–[24], and a high specificity (99–100%).[25] Because the C6 ELISA does not distinguish between IgG and IgM antibodies, in-house IgM and IgG immunoblots were used concurrently as described [26]. Reactions to the 15–20, 22, 30–39, and 41 kDa bands were evaluated for the IgM immunoblot and reactivity to the 22 kDa band with at least one other band was considered as a positive result. The IgM immunoblot was considered borderline if there was a reaction with two or more bands but not the 22 kDa. For the IgG immunoblot, reactions to the 17, 22, 31, 34, 39, 41, 58 and 92 kDa band were evaluated. The IgG immunoblot was considered positive if at least four reactive bands were present including at least one of the following specific bands: 17, 22, 39, 58 and 92 kDa. The IgG immunoblot was considered borderline when four or more bands were present but none of the 17, 22, 39, 58 or 92 kDa bands reacted or when three bands, including at least one specific band, were present. All other results were considered negative.

Seroconversion of IgM or IgG in the immunoblot or in the C6 ELISA was considered as evidence of an early *Borrelia* infection. For seroconversion in the C6 ELISA, the ELISA index score was required to be <1.10 in the baseline serum, and ≥1.10 in the consecutive serum, with a minimum increase of 1.5 points. A participant was considered serologically negative if no reactivity was detected in both the baseline and consecutive serum sample, with any of the serological tests. For analysis of the risk of *Borrelia* infection after a tick bite, borderline results in the C6 ELISA and immunoblot were considered negative. Two consecutive positive serological outcomes without significant in- or decrease of antibody levels and seroreversion (a positive result in the baseline serum sample that became negative on follow-up) were not considered as recent *Borrelia* infections related to the tick consult at enrolment in our analyses. Although serological testing is not recommended after a tick bite or EM, individual serological results were reported to the general practitioner when the paired serology indicated a recent infection, if the case had given written permission for this on the informed consent form.

### Statistical analyses

Statistical analyses were performed with SAS 9.3 (SAS Inc.). The outcome measure “*Borrelia* infection within the three-month follow-up period after a tick bite” was defined as development of physician-confirmed clinical Lyme borreliosis such as EM, or seroconversion for *Borrelia*-specific antibodies. The risk of *Borrelia* infection after a tick bite was estimated with 95% confidence intervals based on mid-P exact. Several multivariate models were developed using logistic regression. All possible predictive variables were included in the multivariate logistic regression models, after which the models were optimized using backwards elimination, until all predictive variables that were maintained in the model were statistically significant contributors (p<0.05). Two separate logistic regression models were developed to identify distinctive symptoms at baseline and three months later at follow-up, for both the tick bite case group and the EM case group. The models compared symptoms at baseline to symptoms at follow-up within each individual case. In a further logistic regression model, adjusted for age, the reported symptoms of EM cases at baseline were compared to those of tick bite cases at baseline, in order to identify differences in symptoms at baseline. A fourth logistic regression model was developed to pinpoint symptoms that are indicative of *Borrelia* infection, other than the pathognomic EM. In this model, the development of new symptoms since baseline were compared between the cases that developed *Borrelia* infection after a tick bite, and those that did not develop EM or seroconverted after a tick bite. Logistic regression was also applied to identify possible predictors of *Borrelia* infection after a tick bite, adjusted for age. For this analysis, the development of EM or seroconversion were tested for associations with tick infection with *Borrelia*, tick engorgement, and tick attachment time reported by the participant. The same analysis was performed to look for predictors of symptoms that could be indicative of *Borrelia* infection, as identified in the earlier described fourth model on symptoms. Tick bite cases that received (prophylactic) antibiotic treatment for a tick bite at enrolment, were excluded from the analyses of the risk of *Borrelia* infection and other reported symptoms after a tick bite, as well as one case whose development of neuroborreliosis was almost certainly not attributable to the tick bite at baseline case #15 in table 1 and 2).

**Table 1:** Clinical manifestations of Lyme borreliosis, serology and exposure to ticks among tick bite cases with evidence of *Borrelia* infection within three months after a tick bite.

Case	Evidence of *Borrelia* infection		C6 ELISA index*		IgM immunoblot		IgG immunoblot		*Borrelia* in tick	Tick engorgement	hours of tick attachment
	Clinical	Seroconversion**	1st	2nd	1st	2nd	1^st^	2^nd^			
1	yes (EM)	yes (IgG)	1.12	0.83	−	−	+/−	+	n.a.	n.a.	30
2	yes (EM)	yes (C6)	0.21	1.90	−	−	−	−	+ (untypeable *Borrelia*)	full	16
3	yes (EM)	no	0.49	0.52	+/−	+/−	−	−	+ *(B. afzelii)*	unengorged	60
4	yes (EM)	no	0.27	0.37	+/−	+/−	−	−	−	full	36
5	yes (EM)	no	0.47	0.43	−	−	−	−	n.a.	n.a.	8
6	yes (EM)	no	0.64	0.98	+/−	+/−	−	−	−	unengorged	30
7	yes (EM)	no	0.37	0.33	−	−	−	−	+ (untypeable *Borrelia*)	partial	15
8	no	yes (C6 & IgM & IgG)	0.40	3.79	−	+	−	+	n.a.	n.a.	26
9	no	yes (C6)	0.29	3.69	+/−	+/−	−	−	n.a.	n.a.	20
10	no	yes (C6 & IgM)	0.37	2.63	−	+	−	+/−	n.a.	n.a.	24
11	no	yes (C6 & IgM)	0.76	6.57	+/−	+	−	+/−	+ (untypeable *Borrelia*)	full	20
12	no	yes (C6)	0.42	7.01	−	+/−	−	+/−	−	partial	n.a.
13	no	yes (C6)	0.22	1.86	−	−	−	−	+ (*B. afzelii*)	partial	n.a.
14	no	yes (C6 & IgG)	0.26	5.84	−	−	−	+	+ (untypeable *Borrelia*)	partial	72
15 ‡	yes (neuroborreliosis)	no	4.12	6.49	+	+	+	+	−	Full	36

EM  =  erythema migrans; n.a.  =  no tick available for testing.

* C6 ELISA test results were scored as negative (ELISA index score, <0.90), borderline (0.90 to 1.09), or positive (≥1.10).

** Seroconversion of IgM and/or IgG in the immunoblot and/or in the C6 ELISA was considered as evidence of an early *Borrelia* infection. For seroconversion in the C6 ELISA, the ELISA index score was required to be <1.10 in the baseline serum, and ≥1.10 in the consecutive serum, with a minimum increase of 1.5 points.

‡ case #15 was excluded from risk analyses, as the development of Lyme borreliosis was almost certainly not attributable to the tick bite at baseline.

**Table 2:** Self-reported symptoms among cases with evidence of *Borrelia* infection within three months after a tick bite.

Case	Symptoms at baseline	Symptoms at follow-up
1	rash other than EM	EM
2	no	EM
3	no	EM, headache, impaired concentration, elevated body temperature, myalgia, pain in limbs, weight increase of 4 kilograms
4	no	EM, elevated body temperature, tingling sensation in limbs
5	rash other than EM	EM
6	no	EM
7	no	EM, joint pain, tingling sensation in limbs, blurred vision, loss of power, cold hands and feet
8	no	rash other than EM, itching at tick bite site
9	no	No
10	no	Headache
11	no	No
12	not available	not available
13	no	rash other than EM, swollen tick bite site, headache, elevated body temperature, myalgia, joint pain, pain in limbs, abdominal pain
14	no	rash other than EM, itching at tick bite site
15 ‡	no	neuroborreliosis, manifest as a Bell's palsy (one-sided facial paralysis), rash other than EM, headache, myalgia, joint pain, pain in limbs, gastro-intestinal complaints

EM  =  erythema migrans.

‡ case #15 was excluded from risk analyses, as the development of Lyme borreliosis was almost certainly not attributable to the tick bite at baseline.

## Results

### Study population

**Figure 1:**
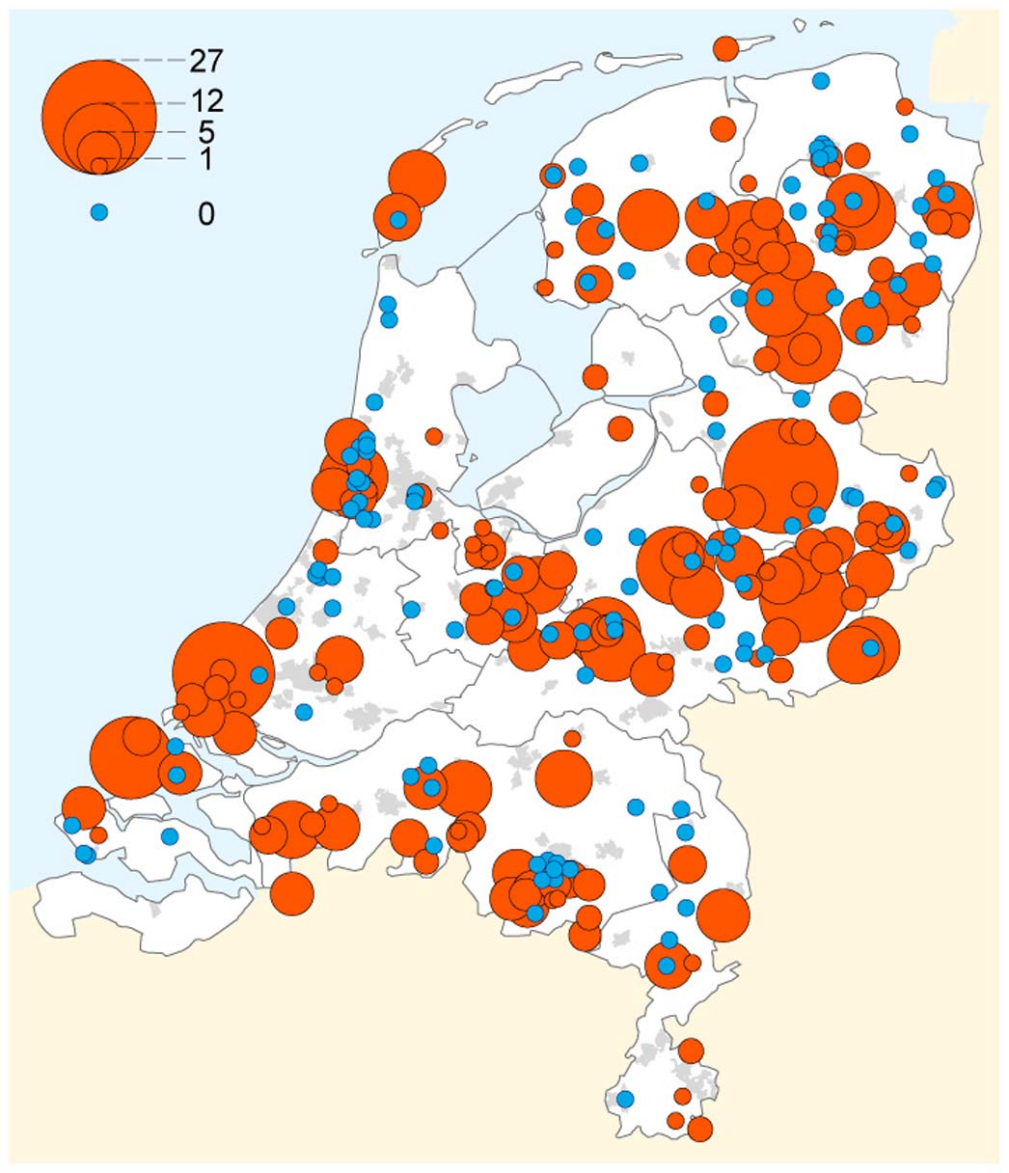
Geographical distribution of 644 cases with tick bites or erythema migrans that participated in the study, depicted as the number of cases per selected general practitioner's practice.

**Figure 2:**
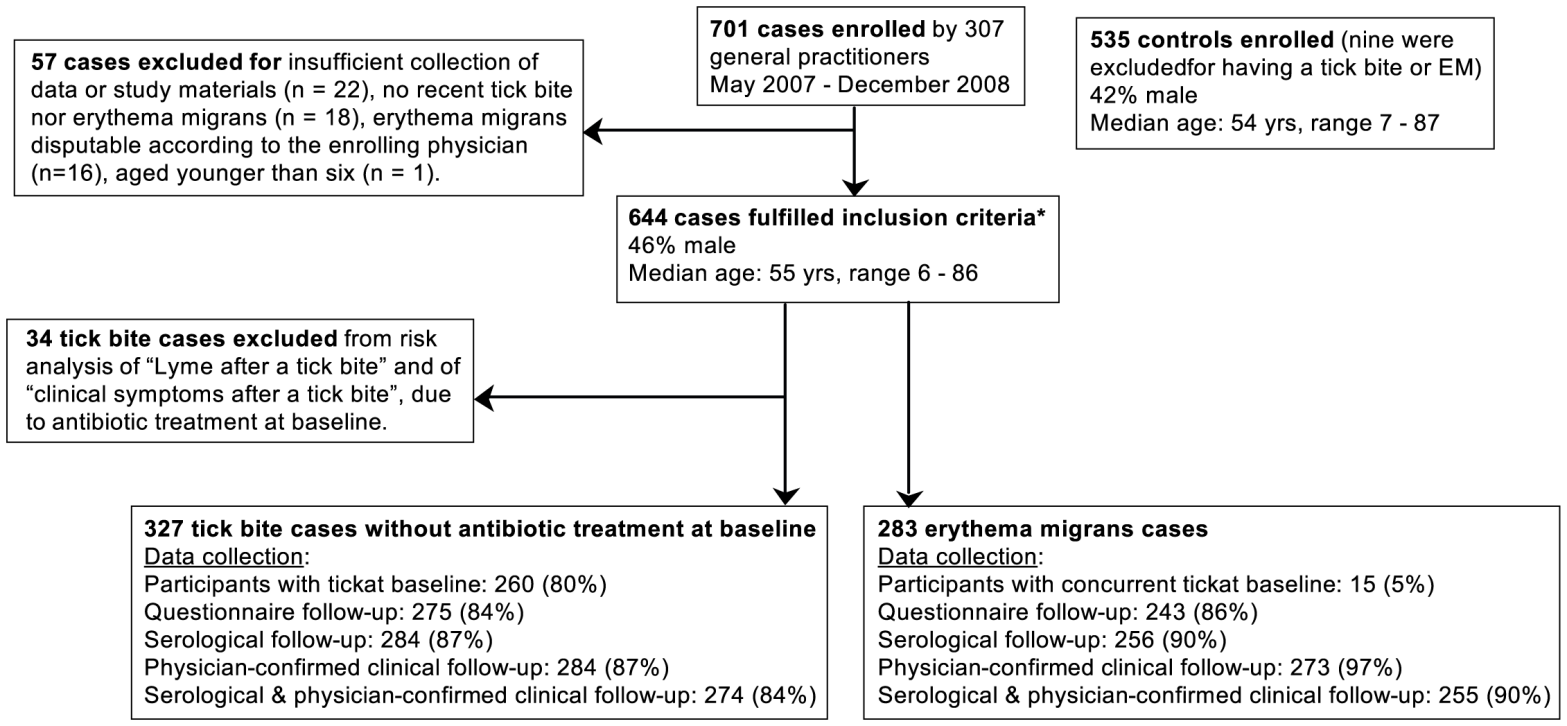
Flow chart of participants and collection of study materials. * Inclusion criteria: (a) the patient consulted one of the cooperating GP's for a recent tick bite or an erythema migrans, and (b) the patient was at least six years of age, and (c) the tick bite had occurred within the Netherlands.

Of the 307 cooperating general practitioners, 180 (59%) provided one or more participants. A total of 644 participants were evenly distributed over the Netherlands (figure 1), but concentrated in areas with a high incidence of tick bites, due to the selection of invited general practitioners. The median patient enrolment was 15% per physician for tick bite patients and 33% per physician for EM patients, based on weekly scores of 90 general practitioners. A flow chart of participants is shown in figure 2, with demographic characteristics and data collection on submitted ticks and follow-up of questionnaires, serology, and physician-confirmed clinical outcomes. Among the 644 participants, 361 cases (55%) consulted their physician for a tick bite, and 283 (43%) consulted their physician at baseline with an EM that was undisputed by their physician.

### Tick analyses

Three hundred and fourteen ticks were obtained from 293 participants. The majority (94%) of these ticks were identified as *Ixodes ricinus*. Eighteen ticks (6%) could not be identified, as they had been damaged too much during removal from the patient's skin. Of these participants who submitted ticks, 278 were cases who consulted their physician for a tick bite, and fifteen were cases who consulted their physician with an EM. Among the EM cases who submitted a tick, seven had saved the tick that had been removed earlier, and in six cases the tick was still attached at the site of the EM, as confirmed by the physician. Two EM cases submitted a tick that was removed by the general practitioner found on a different location on the body. The majority of tick bite cases submitted one tick (94%), and seventeen cases submitted more than two, up to five ticks.

Table 3 shows developmental stage of the ticks, degree of engorgement and the *Borrelia* species detected in these ticks. *Borrelia*-positive ticks per developmental stage and engorgement are shown in supplementary table S1. *Borrelia burgdorferi* sensu lato DNA was detected in 92 out of 314 ticks (29.3% (95%CI: 24.5%–34.5%). Four different species of *Borrelia* were identified among which *B. afzelii* predominated (11.5%), followed by *B. garinii* (3.5%), *B. burgdorferi* sensu stricto (ss.) (2.2%), and *B. valaisiana* (1.3%). In one tick *B. burgdorferi* ss. and *B. garinii* were detected simultaneously, and another tick contained *B. garinii* and *B. afzelii*. Thirty-six ticks (11.5%) contained *B. burgdorferi* sensu lato, which could not be typed further.

**Table 3:** Characteristics of 314 ticks obtained from 293 participants.

	n	*% of ticks*	*(%) of species per genus*
**Developmental stage**			
Larva	4	*1.3%*	
Nymph	167	*53.2%*	
Adult	135	*43.0%*	
Not identified	8	*2.6%*	
**Engorgement**			
Unengorged	110	*35.0%*	
Partially engorged	114	*36.3%*	
Fully engorged	64	*20.4%*	
Not determined	26	*8.3%*	
**Detected DNA sequences** *			
* Borrelia* spp	92	*29.3%*	
* B. afzelii*	36	*11.5%*	*(38.2%)*
* B. garinii*	11	*3.5%*	*(11.7%)*
* B. burgdorferi* sensu stricto	7	*2.2%*	*(7.4%)*
* B. valaisiana*	4	*1.3%*	*(4.2%)*
untypeable *Borrelia*	36	*11.5%*	*(38.2%)*

* See supplemental table S1 for *Borrelia*-positive ticks by developmental stage and engorgement of 314 ticks obtained from 293 participants.

### Follow-up of cases with a tick bite at baseline

**Figure 3:**
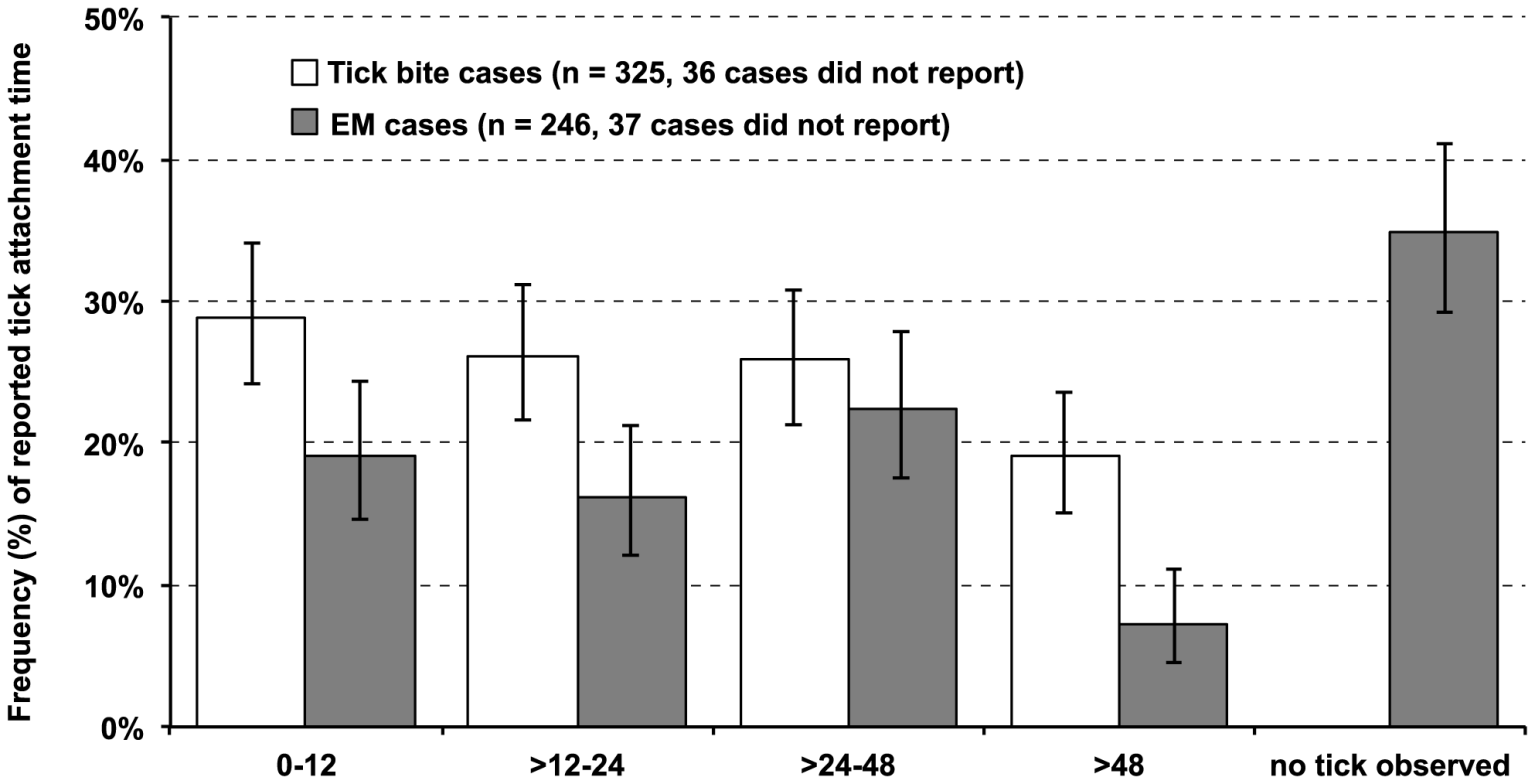
Frequencies of reported tick attachment times from 361 tick bite cases and 283 erythema migrans cases, of which respectively 36 and 37 cases did not report attachment time.

**Figure 4:**
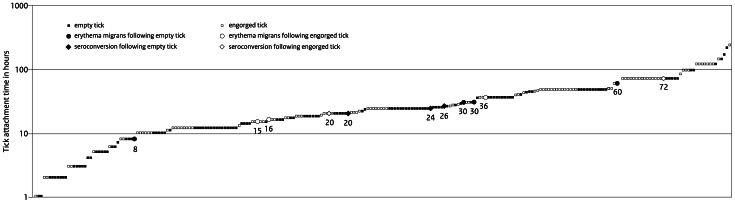
Ranked scatter plot of tick attachment times from 274 tick bite cases that did not receive antibiotics at baseline, excluding twenty cases (7%) that did not report tick attachment time. Fourteen cases had evidence of early *Borrelia* infection (erythema migrans or seroconversion) within three months after a tick bite, of which two cases did not report attachment time.

**Figure 5:**
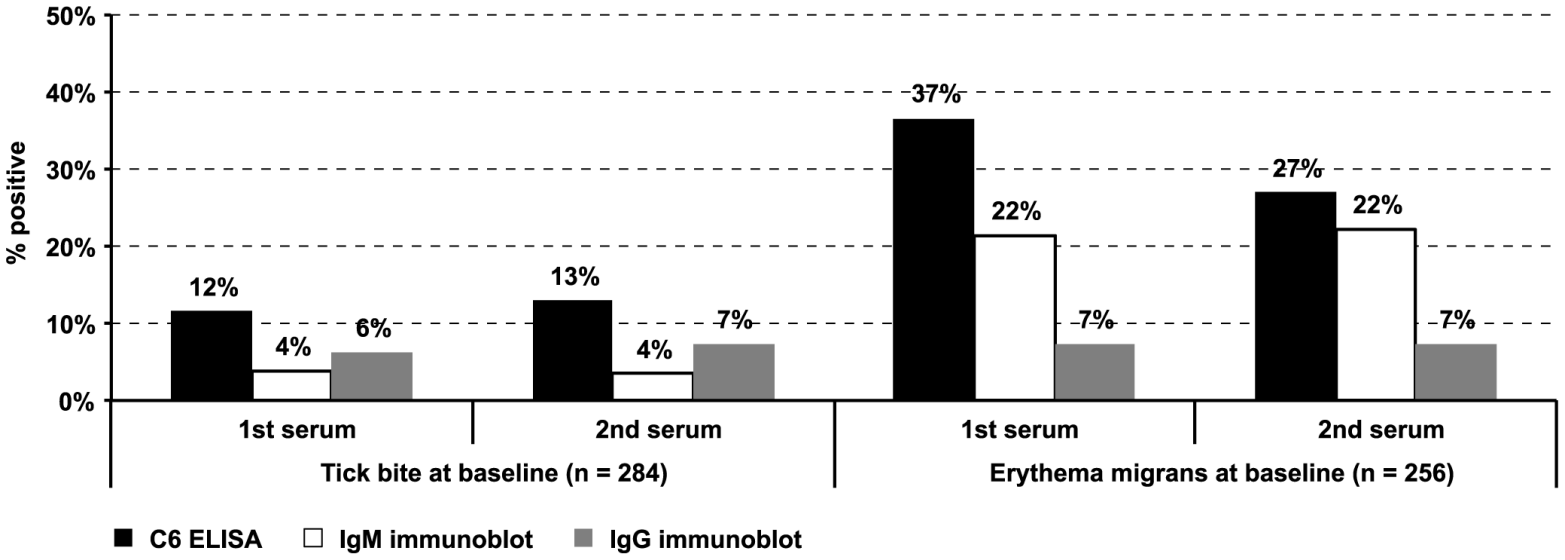
Seropositive results of the C6 ELISA*, the IgM** and IgG*** immunoblot for *Borrelia*-specific antibodies in simultaneously tested paired serum samples, collected at enrolment and at follow-up after three months. * The C6 ELISA test was considered positive if the ELISA index scored ≥1.10.** The IgM immunoblot was considered positive if there was reactivity to the 22 kDa band, together with at least one of the following specific bands: 15–20, 22, 30–39, and 41 kDa.*** The IgG immunoblot was considered positive if at least four reactive bands were present including at least one of the following specific bands: 17, 22, 39, 58 and 92 kDa.

Among the 361 cases that consulted their physician for a tick bite, 34 (9%) had received antibiotics at baseline, even though the national medical guidelines did not recommend prophylactic treatment after a tick bite [27]. None of these 34 tick bite cases with antibiotics at baseline, reported development of clinical Lyme borreliosis, even though six out of eighteen corresponding ticks tested positive for *Borrelia*. Reported tick attachment times ranged between one hour and sixteen days. More than half of the tick bite cases (56%) reported that the tick had been removed within 24 hours (figure 3). Figure 4 shows reported tick attachment times and development of early *Borrelia* infection for the 327 tick bite cases that did not receive antibiotic treatment at baseline. Seventeen percent of the tick bite cases reported other tick bites during the six weeks before enrolment or within the three-month follow-up. Among these cases that reported other tick bites during this period before or after enrolment, the majority (44/54) reported one to three tick bites, but some (10/54) reported more than three, and up to sixteen tick bites. Seropositive outcomes of all cases, for the C6 ELISA, the IgM and IgG immunoblot are shown in figure 5. The majority of the 284 tick bite cases with serological follow-up (85.9%) tested negative for *Borrelia*-specific antibodies at baseline and remained negative at follow-up in the immunoblots as well as in the C6-ELISA. Thirty-one tick bite cases (10.9%) tested positive for *Borrelia*-specific antibodies at baseline and at follow-up. Seroconversion for *Borrelia*-specific antibodies was observed in nine tick bite cases (3.2%) with any of the three tests, and in five tick bite cases (1.8%) confirmed with the immunoblot following a positive or borderline outcome in the ELISA. Within the follow-up period of three months after a tick bite, fourteen cases had evidence of an early *Borrelia* infection, clinically and/or serologically. Seven cases developed EM, of which two cases also seroconverted. Additionally, seven other cases had serological evidence of an early *Borrelia* infection. For the cases with *Borrelia* infection after a tick bite, table 1 shows the clinical manifestations of Lyme borreliosis, serology and exposure to ticks, and table 2 shows their self-reported symptoms at baseline and three months later at follow-up. Table 4 shows symptoms reported by all cases at baseline and three months later at follow-up. These symptoms at baseline and three months later at follow-up were compared in a multivariate logistic regression model, matched by case. The only symptom with a statistically significant different frequency at baseline compared to follow-up among tick bite cases, was an indistinct rash at the tick bite site, which occurred among 12.3% of these cases at baseline and 7.1% at follow-up. The emergence of new symptoms since baseline after a tick bite among cases with seroconversion or EM were compared in another multivariate logistic regression model, to the tick bite cases that did not have *Borrelia* infection. Tick bite cases with seroconversion or EM were more likely to have newly emerged symptoms such as headache, tingling sensations in limbs, and itching (table 4).

**Table 4:** Multivariate logistic regression analyses of symptoms reported by tick bite cases and erythema migrans (EM) cases.

	Symptoms of tick bite cases										Symptoms of EM cases								
	at baseline (n = 316)		at follow-up (n = 297)		Newly emerged among cases with:						at baseline (n = 277)		at follow-up (n = 240)		EM cases at follow-up versus baseline *		EM cases at baseline versus tick bite cases at baseline †		
					*Borrelia* infection (n = 13)		no *Borrelia* infection (n = 259)												
	n	%	n	%	n	%	n	*%*	mOR	(95%CI) p-value	n	%	n	%	mOR	(95%CI) p-value	mOR	(95%CI) p-value	
rash other than EM	39	*12.3*	20	*6.7* ‡	3	*23.0*	11	*4.2*	ns		28	*10.1*	15	*6.3*	0.4	(0.2–1.0) p = 0.04	ns		
headache	33	*10.4*	23	*7.7*	3	*23.0*	8	*3.1*	14.2	(2.7–74.7) p = 0.01	61	*22.0*	43	*17.9*	0.4	(0.2–0.8) p = 0.01	2.6	(1.6–4.2) p<0.001	
vomiting and nausea	4	*1.3*	7	*2.4*	0	*0*	3	*1.2*	∼		2	*0.7*	3	*1.3*	ns		ns		
impaired concentration	8	*2.5*	10	*3.4*	1	*7.7*	6	*2.3*	ns		18	*6.5*	20	*8.3*	6.4	(1.5–26.2) p = 0.01	ns		
elevated body temperature	10	*3.2*	13	*4.4*	3	*23.0*	5	*1.9*	ns		19	*6.9*	14	*5.8*	ns		ns		
myalgia	34	*10.8*	26	*8.8*	2	*15.4*	12	*4.6*	ns		48	*17.3*	35	*14.6*	ns		ns		
joint pain	25	*7.9*	30	*10.1*	2	*15.4*	18	*6.9*	ns		41	*14.8*	33	*13.8*	ns		ns		
blurred sight	6	*1.9*	9	*3.0*	1	*7.7*	4	*1.5*	ns		8	*2.9*	7	*2.9*	ns		ns		
tingling sensation in limbs	19	*6.0*	15	*5.0*	2	*15.4*	8	*3.1*	14.8	(2.3–93.7) p = 0.01	30	*10.8*	23	*9.6*	ns		ns		
pain in limbs	18	*5.7*	18	*6.1*	2	*15.4*	8	*3.1*	ns		28	*10.1*	23	*9.6*	ns		ns		
(partial) facial paralysis	0	*0*	1	*0.3*	0	*0*	1	*0.4*	∼		0	*0*	0	*0*	∼		∼		
itching	3	*1.0*	6	*2.0*	2	*15.4*	2	*0.8*	32.5	(3.8–278.6) p = 0.002	7	*2.5*	6	*2.5*	ns		ns		
tiredness	7	*2.2*	5	*1.7*	1	*7.7*	2	*0.8*	ns		11	*4.0*	11	*4.6*	ns		ns		
dizziness	0	*0*	1	*0.3*	0	*0*	1	*0.4*	∼		4	*1.4*	4	*1.7*	∼		∼		
swollen tick bite site	2	*0.6*	4	*1.4*	0	*0*	3	*1.2*	∼		6	*2.2*	0	*0*	ns		ns		

EM  =  erythema migrans; mOR  =  multivariate odds ratio; CI  =  confidence interval; ns  =  not statistically significant.

* multivariate logistic regression model, matched by case.

† multivariate logistic regression model, adjusted for age.

‡ significantly lower risk of an indistinct rash (other than EM) at follow-up than at baseline in the multivariate logistic regression model, matched by case (odds ratio 0.5, 95%CI: 0.3–0.9, p-value 0.03).

### Risk of *Borrelia* infection after a tick bite

Among 274 tick bite cases with serological follow-up and physician-confirmed clinical follow-up, fourteen cases had evidence of an early *Borrelia* infection, of which seven cases developed EM (table 1). This yields an estimated risk of 2.6% (95%CI: 1.1%–5.0%) for development of EM within three months after a tick bite. For development of either EM or seroconversion, the risk was 5.1% (95%CI: 2.9%–8.2%). Table 5 shows early *Borrelia* infection after a tick bite associated with tick infection with *Borrelia*, tick engorgement and tick attachment time reported by the participant. Among cases with a *Borrelia*-positive tick, the risk for development of an EM was 4.4%, the risk of seroconversion was 5.9%, and the risk for development of either EM or seroconversion was 9.0%. Cases with a *Borrelia*-positive tick had a substantially and significantly higher risk of developing EM or seroconversion (odds ratio 4.8, p-value 0.03), and of seroconversion alone (odds ratio 11.1, p-value 0.04). For the development of EM alone, a statistically significant association could not be shown (table 5). Tick engorgement and tick attachment time reported by the participant were not significantly associated with *Borrelia* infection after a tick bite. Additionally, cases with other self-reported symptoms that could be indicative of *Borrelia* infection, such as newly emerged headache, tingling sensation in limbs, or itching (as observed in table 4), were analyzed as an outcome group together with the cases that developed EM or seroconversion. This analysis did not yield any statistically significant associations with tick infection with *Borrelia*, tick engorgement or tick attachment time.

**Table 5:** Predictors of *Borrelia* infection after a tick bite, among cases that did not receive antibiotics at baseline, using multivariate logistic regression analyses, adjusted for age.

	Developed EM				Developed seroconversion				Developed EM or seroconversion				Developed EM or seroconversion or newly emerged headache, tingling sensations, or itching			
	Yes (n = 7)		No (n = 276)		Yes (n = 9)		No (n = 274)		Yes (n = 14)		No (n = 259)		Yes (n = 32)		No (n = 241)	
	n	*%*	n	*%*	n	*%*	n	*%*	n	*%*	n	*%*	n	*%*	n	*%*
***Borrelia*** ** detected in tick**																
*mOR (95%CI), adjusted for age*	*OR: ns*				*OR: 11.1 (1.1–108.9), p = 0.04*				*OR: 4.8 (1.1*–*20.4), p = 0.03*				*OR: ns*			
No	2	*1.3*	154	*98.7*	1	*0.6*	154	*99.4*	3	*2.0*	146	*98.0*	13	*8.7*	136	*91.3*
Yes	3	*4.4*	65	*95.6*	4	*5.9*	64	*94.1*	6	*9.0*	61	*91.0*	11	*16.4*	56	*83.6*
No tick collected	2		57		4		56		5		52		8		49	
**Engorgement of the tick**																
*mOR (95%CI), adjusted for age*	*OR: ns*				*OR: ns*				*OR: ns*				*OR: ns*			
Unengorged	2	*2.6*	75	*97.4*	0	*0*	78	*100*	2	*2.7*	72	*97.3*	8	*10.8*	66	*89.2*
Engorged	3	*2.3*	126	*97.7*	5	*3.9*	123	*96.1*	7	*5.6*	119	*94.4*	15	*11.9*	111	*88.1*
Not determined	2		75		4		73		5		68		9		64	
**Duration of tick attachment**																
*mOR (95%CI), adjusted for age*	*OR: ns*				*OR: ns*				*OR: ns*				*OR: ns*			
Within 24 hours	3	*1.9*	156	*98.1*	4	*2.5*	155	*97.5*	6	*3.9*	147	*96.1*	20	*13.1*	133	*86.9*
More than 24 hours	4	*3.5*	109	*96.5*	3	*2.7*	110	*97.3*	6	*5.5*	103	*94.5*	10	*9.2*	99	*90.8*
Unknown	0		11		2		9		2		11		2		9	

EM  =  erythema migrans; mOR  =  multivariate odds ratio; CI  =  confidence interval; ns  =  not statistically significant.

### Follow-up of cases with erythema migrans at baseline

Roughly two third (66%) of the 283 cases that consulted their physician for an EM at baseline, reported that they had noticed a tick prior to their EM. Among the EM cases that had noticed a tick bite, the reported durations of tick attachment varied between one hour and fourteen days. Half of these cases (55%) reported that the tick had been removed within 24 hours (figure 3). All cases with EM were treated with antibiotics at baseline, in accordance with the national medical guidelines for treatment of Lyme borreliosis [27]. Seropositive outcomes of the C6 ELISA, the IgM and IgG immunoblots for *Borrelia*-specific antibodies in simultaneously tested paired serum samples of all cases are shown in figure 5. Among 256 EM cases with serological follow-up, the majority (63.3%) tested negative for *Borrelia*-specific antibodies at baseline and remained negative at follow-up in the immunoblot as well as in the C6-ELISA. Fourteen EM cases (5.5%) had serological evidence of an early *Borrelia* infection. Twelve of these cases seroconverted only in the IgM immunoblot, one case seroconverted in the IgG immunoblot and the C6 ELISA, and one case seroconverted only in the C6 ELISA. Seventy-three EM cases (28.5%) tested positive for *Borrelia*-specific antibodies at baseline and at follow-up. Seroreversion was observed in seven cases (2.7%), meaning that the baseline serum tested positive and the follow-up serum tested negative, which may be caused by antibiotic treatment for EM [28]. Self-reported symptoms during the preceding two weeks, as reported by EM cases at baseline and follow-up, are presented in table 4. Among the 273 EM cases with physician-confirmed clinical follow-up, three cases (1%) reported persisting symptoms at three months follow-up, after antibiotic treatment for EM at baseline. One case was referred to the neurologist for persisting myalgia, joint pain and headache. A second case reported tingling sensations, and continuing expansion of the EM despite treatment. The third case reported persisting flu like symptoms. Both the second and third case received additional antibiotic treatment, two weeks after initial treatment for the EM from their general practitioner.

The analysis of self-reported symptoms reported by EM cases at baseline and at three months follow-up (table 4) showed a statistically significantly higher frequency of headache at baseline (22.0%) than at follow-up (17.9%), and a higher frequency of an indistinct rash at the tick bite site at baseline (10.1%) than at follow-up (6.3%), and a lower frequency of impaired concentration at baseline (6.5%) than at follow-up (8.3%). For the other reported symptoms, no statistically significant differences were found between baseline and follow-up. A comparison at baseline of EM cases with tick bite cases shows that EM cases report headache more often (22.0%) than tick bite cases (10.4%). For the other reported symptoms, no statistically significant differences were found between EM cases and tick bite cases at baseline.

## Discussion

As the incidence of general practitioner consultations for tick bites and EM in the Netherlands have increased markedly during the past decade [2]–[4], understanding and quantification of the risk of infection after a tick bite are required. In the current study, one out of forty participants (7/274 = 2.6%) developed an EM within three months after a tick bite, which can be considered as a substantial risk. This was under the conditions that 29% of the ticks tested positive for *Borrelia*, and 57% of the subjects reported tick removal within 24 hours. A lower risk of 0.7% (1/146) was estimated by Jacobs *et al.* from a smaller study between 2004 and 2006 in the Netherlands. However, their tick infection rate was lower (20%), and the majority (84%) of their subjects reported tick removal within 24 hours [17]. According to similar studies performed during the past decade in Western Europe, risk estimates for development of EM after a tick bite vary between 0.3% (1/341) in Sweden [16], 0.8% (3/376) in Switzerland [14], and 5.2% (14/269) in Switzerland [15]. Risk estimates within this range are also observed in the United States. According to a meta-analysis of four clinical trials on antibiotic prophylaxis for the prevention of Lyme borreliosis, performed in the North Eastern states of Connecticut and New York, the pooled risk of Lyme borreliosis after an *Ixodes scapularis* tick bite was 2.2% (12/539), without prophylactic antibiotics [26]. Although prophylactic antibiotic treatment can prevent most Lyme borreliosis after detected tick bites [11], the high NNT poses a substantial disadvantage. Aiming to explore ways to reduce the NNT, we investigated tick-screening instruments such as tick testing for infection with *Borrelia* and assessment of the duration of the tick's blood meal. Our data suggest that tick testing for *Borrelia* infection may be useful in the assessment of an individual's risk, as we observed a statistically significant higher risk of developing EM or seroconversion combined and of seroconversion alone, among cases with a *Borrelia*-positive tick (odds ratio 4.8 and 11.1 respectively, table 5). The risk for development of EM alone was elevated, but did not reach statistical significance in our logistic regression model. Tick engorgement and self-estimated tick attachment time also yielded elevated but non-significant risks for *Borrelia* infection in our logistic regression model. Non-significantly elevated risk estimates for *Borrelia*-positive ticks, and for longer duration of tick attachment were also reported from other studies on the risk for development of *Borrelia* infection [14]–[16]. To some extent, this lack of statistically significant predictors of *Borrelia* infection may be due to insufficient numbers of enrolled cases or insufficient accuracy of the measures. During the spring of 2013, we started a randomized controlled intervention study, investigating the efficacy of prophylactic antibiotic treatment after a tick bite. The rationale for this effort, supplementary to the outcomes of North American studies [11], will be that Europe has different transmission dynamics than North America, as Lyme borreliosis in Europe is transmitted by *Ixodes ricinus, and* caused mainly by other species from the *Borrelia burgdorferi* sensu lato group, such as *B. afzelii* and *B. garini*. Through our tick bite notification website [29], we aim to enroll approximately 2500 tick bitten participants within four years for this upcoming nationwide study on the efficacy of prophylaxis after a tick bite, randomly assigning the tick bite participants to a treated and untreated group. This amount of participants should provide sufficient power to assess the extent to which NNT can be reduced by tick screening criteria, for instance only prescribing prophylaxis if the tick is infected and if tick engorgement is above a certain threshold.

Further understanding is also needed on the development of *Borrelia* infection after the bite of a tick that tested negative for *Borrelia*, or after short tick attachment duration. We observed *Borrelia* infection in 2% (3/149) of the cases with a *Borrelia*-negative tick, in 3% (2/74) of our cases with a tick bite with a low degree of tick engorgement, and in 4% (6/153) of the tick bite cases who reported an attachment duration below the 24-hours tick-to-host transmission threshold for *Borrelia* (table 5). One explanation for development of *Borrelia* infection after the bite of a *Borrelia*-negative tick, may be that these ticks contained *Borrelia* species that are not detected by our assays, because the assays were not sensitive enough or because the human blood meal present in some ticks could have inhibited the PCR. However, since the infection rates were alike among ticks with different degrees of engorgement, the blood meal probably did not inhibit the PCR. Another hypothesis could be that the tick may have injected the major portion or even all of its bacterial charge during the blood meal. In addition, we cannot exclude the possibility of incorrect diagnosis of EM by the physician in two of these three cases with a *Borrelia*-negative tick, who did not seroconvert but did report an EM at follow-up. Nevertheless, infection may have been transmitted through a different tick bite, which may have gone unnoticed. Other tick bites shortly before or during the follow-up period were reported by 1/3 cases who developed *Borrelia* infection after the bite of a *Borrelia*-negative tick, 1/6 cases with a <24-hours tick attachment time, and 0/2 cases with a tick bite with a low degree of engorgement. However, it is estimated that one third, up to two thirds of tick bites go unnoticed [12], [13]. Accordingly, 34% of the cases who enrolled with an EM in our study did not recall a preceding tick bite. Tick attachment times shorter than 24 hours were also reported by half of our 246 cases that enrolled with an EM and recalled a tick bite shortly before the EM (figure 3). Although the reliability of self-estimated tick attachment time is difficult to assess, our observations underscore the possibility of *Borrelia* transmission when tick attachment duration is shorter than 24 hours.

The proportion of *Borrelia* species identified in *Borrelia*-positive ticks from our tick bite cases was similar to reports on *Borrelia* species identified in field ticks in the Netherlands [19]. Among the six cases who developed *Borrelia* infection after the bite of a *Borrelia*-positive tick, the *Borrelia* species from 2/6 ticks were identified as *B. afzelii*, and 4/6 ticks contained *B. burgdorferi* sensu lato, which could not be typed further (table 1). Proportions of identified *Borrelia* species did not differ with statistical significance between ticks of cases who developed *Borrelia* infection and *Borrelia*-positive ticks of cases who did not develop *Borrelia* infection (results not shown). Neither was there sufficient statistical power to investigate associations between *Borrelia* species and symptoms.

The rising incidence of tick bites and *Borrelia* infections in the Netherlands, poses a considerable threat to public health. However, there is also substantial exposure to other tick-borne microorganisms, as ticks in the Netherlands can also be infected with a wide variety of established or potentially pathogenic microorganisms, such as *Anaplasma phagocytophilum*, Noehrlichia mikurensis, *Rickettsia helvetica*, and *Babesia microti* [19], [30]–[32]. Even though none of our tick bite cases reported acute overt symptoms that would indicate a corresponding illness, we plan further analyses for tick infection with *Ehrlichia, Anaplasma*, *Rickettsia*, *Babesia*, and *Bartonella* species, serological evidence of exposure, and associations with symptoms.

A comparison of self-reported symptoms in our study showed that EM cases were more likely to report headache at baseline than tick bite cases (table 4). Within the group of EM cases, the frequencies of headache and of indistinct rashes were higher at baseline than at follow-up, and there was a lower frequency of impaired concentration at baseline than at follow-up. Furthermore, tick bite cases with early *Borrelia* infection were more likely to have newly emerged symptoms such as headache, tingling sensations in limbs, and itching (table 4). These observations are in line with other studies reporting symptoms associated with early Lyme borreliosis, which are mainly non-specific and frequent in the general population [13], [33], [34]. Our cases were asked to report their symptoms through the questionnaires at baseline and at follow-up. Confirmation by the physician was requested for all reported clinical manifestations of Lyme borreliosis, but as the other self-reported symptoms were not verified, these data should be interpreted with some caution. Remarkably, fifteen cases (5%) who enrolled with an EM also provided a tick at baseline (figure 2). Seven of these patients were enrolled and confirmed later by the physician as EM case, having a concurrent tick bite at baseline. The remaining eight EM cases presented an earlier removed tick, which they had stored to bring it along for the physician. As we did not provide the general practitioners with a case-definition for EM, the confirmation of EM was based on the physicians' expertise and ability to discriminate an EM from other types of rashes.

EM was the only physician-confirmed clinical sign of Lyme borreliosis observed in this study. Tick bite cases and their physicians did not report early neuroborreliosis or borrelial lymphocytoma that could be related to the tick bite at enrolment. As our study comprised a follow-up period of three months after the tick bite, observing rare events such as disseminated Lyme borreliosis was unlikely.

For epidemiological analysis of the risk of *Borrelia* infection after a tick bite, we deviated from the regular medical serological diagnostic practice, in which serology is normally not recommended after a tick bite or to confirm EM [27]. 28.5% of our EM cases and 10.9% of tick bite cases tested positive for *Borrelia*-specific antibodies at baseline and at follow-up after three months without a clear in- or decrease of antibody levels, which provided no discrimination between recent and old *Borrelia* infection. Among our EM cases, only 5.5% seroconverted, and seroreversion was observed in seven 2.7%, meaning that the baseline serum tested positive and the follow-up serum tested negative. Most likely antibiotic treatment for EM may have influenced the development of an antibody response [28]. This illustrates the low sensitivity of serology in the early stages of Lyme borreliosis [35], which is why serology is not recommended after a tick bite or to confirm EM. In regular medical serological diagnostic practice, a positive or borderline ELISA assay outcome requires confirmation by IgM or IgG immunoblot. However, in our study seroconversion with any of these assays was considered evidence of an early *Borrelia* infection. Seroconversion in the C6 ELISA, without confirmation in the Immunoblot, was observed in one tick bite case who developed EM (case#2 in table 1), and in three tick bite cases who did not develop EM within the follow-up period (case#9, #12, #13 in table 1). This also occurred in one case who enrolled with an EM (C6 ELISA index score 0.44 at baseline, and 2.64 at follow-up, IgM immunoblot remained borderline and IgG immunoblot remained negative). Antibodies against C6 are of particular diagnostic relevance because they are regarded highly specific (91–100%).[36]–[38] In Europe, the C6 ELISA is reactive in 20–100% of EM patients depending on the duration of the rash. Among the C6 seropositive EM patients however, IgG responses are not always detected in immunoblot indicating that C6 reactivity is an early serological marker for *Borrelia* infection [23], [39].

Patients younger than six years of age were not eligible for participation, due to ethical considerations with regard to the required blood withdrawals for our study. Although there are no nationwide data on the occurrence of tick bites and Lyme borreliosis among children younger than six years of age, this group of young children do not appear to be at high risk of tick bites, according to the reported age-specific occurrence of Lyme borreliosis in the United States [40] and Europe [41], [42]. Therefore, we do not expect that our estimate for the risk of infection after a tick bite will be biased substantially through the exclusion of this age group.

Our study was designed to enroll 1500 participants within one year, which appeared feasible based on expected median numbers of 17.7 tick bite patients per physician and 5.8 EM patients per physician per year, as estimated from earlier questionnaires among all general practitioners in the Netherlands in 2005.[3] Based on the registered number of tick bite patients and EM patients on the weekly scoring cards, we found that half of the eligible patients were not invited.

## Supporting Information

Table S1 ***Borrelia*** **spp. DNA detected in 314 ticks obtained from 293 participants, by developmental stage and engorgement.**
(DOCX)
