# Supplementary material for: A Prospective Study among Patients Presenting at the General Practitioner with a Tick Bite or Erythema Migrans in the Netherlands
Source: PLoS One. 2013 May 16;8(5):e64361. doi: 10.1371/journal.pone.0064361 (PMC3655959; doi:10.1371/journal.pone.0064361)
Supplement: Table S1 — Borrelia spp. DNA detected in 314 ticks obtained from 293 participants, by developmental stage and engorgement. (DOCX) [file pone.0064361.s001.docx]

**Supplementary table 1. *Borrelia* spp. DNA detected in 314 ticks obtained from 293 participants, by developmental stage and engorgement.**

|  |  | **Developmental stage** | | | | |  |  | |  |  | | |  |  | | |
| --- | --- | --- | --- | --- | --- | --- | --- | --- | --- | --- | --- | --- | --- | --- | --- | --- | --- |
|  |  | **Larva (n=4)** | |  | **Nymph (n=167)** | |  | **Adult (n=135)** | |  | **Not identified (n=8)** | | | | **All stages (n=314)** | | |
| **Engorgement** |  | **n^pos^** | ***^stage^%***  ***^engorg^%*** |  | **n^pos^** | ***^stage^%***  ***^engorg^%*** |  | **n^pos^** | ***^stage^%***  ***^engorg^%*** |  | **n^pos^** | ***^stage^%***  ***^engorg^%*** |  | | **n^pos^** | ***^stage^%***  ***^engorg^%*** |  |
| **Unengorged**  **(n=110)** |  | 0 | *-*  *-* |  | 23 | *13.8%*  *20.9%* |  | 10 | *7.4%*  *9.1%* |  | 0 | *-*  *-* |  | | 33 | *30.0%* |  |
| **Partially engorged**  **(n=114)** |  | 0 | *-*  *-* |  | 16 | *9.6%*  *14.0%* |  | 19 | *14.1%*  *16.6%* |  | 0 | *-*  *-* |  | | 35 | *30.7%* |  |
| **Fully engorged**  **(n=64)** |  | 1 | *25.0%*  *1.6%* |  | 6 | *3.6%*  *9.4%* |  | 10 | *7.4%*  *15.6%* |  | 0 | *-*  *-* |  | | 17 | *26.6%* |  |
| **Not determined**  **(n=26)** |  | 0 | *-*  *-* |  | 1 | *0.6%*  *3.8%* |  | 4 | *3.0%*  *15.4%* |  | 2 | *25.0%*  *7.7%* |  | | 7 | *26.9%* |  |
| **All engorgements**  **(n=314)** |  | 1 | *25.0%* |  | 46 | *27.5%* |  | 43 | *31.9%* |  | 2 | *25.0%* |  | | 92 | *29.3%* |  |

n^pos^ = number of *Borrelia*-positive ticks;

^engorg^% = row percentage of *Borrelia*-positive ticks per engorgement;

^stage^% = column percentage of *Borrelia*-positive ticks per developmental stage.
